# Novel opportunity of treatment for psycho-cardiologic disease by gut microbiome

**DOI:** 10.3389/fcvm.2025.1604962

**Published:** 2025-07-22

**Authors:** Yurui Lai, Chenli Fang, Yuang Jiang, Chengying Yang, Qiao Zhou, Yihua Cai, Yan Wei, Xinrong Fan

**Affiliations:** ^1^ Department of Cardiology, The Affiliated Hospital, Southwest Medical University, Luzhou, Sichuan, China; ^2^ Key Laboratory of Medical Electrophysiology, Ministry of Education & Medical Electrophysiological Key Laboratory of Sichuan Province, (Collaborative Innovation Center for Prevention of Cardiovascular Diseases), Institute of Cardiovascular Research, Southwest Medical University, Luzhou, Sichuan, China; ^3^ Department of Cardiology, Institute of Cardiovascular Disease of Chengdu, The Third People’s Hospital of Chengdu, Luzhou, Sichuan, China; ^4^ Department of Cardiovascular Medicine, The Meishan People’s Hospital, Meishan, Sichuan, China

**Keywords:** gut microbiome, metabolites, psycho-cardiologic disease, relationship, treatment

## Abstract

Cardiovascular disease (CVD) patients combined with depression, anxiety, and other psychiatric disorders are becoming a common occurrence. There are many comorbid mechanisms, and CVD patients with psychiatric disorders have poor prognosis. Several studies have shown that dysbiosis and metabolite alterations in the gut were major risk factors for CVD and psychosomatic disorders. This review aims to summarize the mechanisms of gut microbiota and its metabolites in psycho-cardiologic disease, and the therapeutic effects of gut microbiota interventions. It is very useful to propose a new direction for the treatment of psycho-cardiologic disease.

## Introduction

In contemporary clinical practice, the coexistence of cardiovascular disease (CVD) and psychosomatic disorders, often termed “psycho-cardiological disease" (1), is increasingly prevalent (2). Patients with psycho-cardiological disease frequently present with atypical clinical manifestations of CVD and generally experience suboptimal treatment outcomes (3). Moreover, these patients are more prone to exhibit adverse health behaviors, such as poor adherence to treatment, difficulty in breaking harmful habits, and challenge in adopting a healthy lifestyle (4). Conventional treatment approaches for CVD alone are often insufficient, underscoring the growing importance of comprehensively addressing psycho-cardiological disease (5). Consequently, the condition is receiving growing attention from clinicians. Despite extensive basic and clinical research aimed at elucidating the mechanisms underlying this comorbidity and developing targeted therapies—including combined antipsychotic pharmacotherapy, traditional Chinese medicine interventions, cognitive-behavioral therapy, and exercise rehabilitation—the efficacy of these treatments remains limited due to challenges such as drug side effects, variability in treatment efficacy, and limited therapeutic stability. In recent years, there has been growing interest in the role of gut microbiota and its metabolites in the pathophysiology of both CVD and psychosomatic disorders. Thus, the interest has led to the development of concepts such as “gut-heart axis” and “gut-brain axis" (6), which provide a theoretical framework for using gut-targeted interventions in the prevention and treatment of cardiovascular and cerebrovascular diseases. The article reviews current researches on gut microbiota and its metabolites in psycho-cardiological disease, aiming to identify new research directions and potential therapeutic applications.

### Psycho-cardiological disease

The concept of psycho-cardiological disease highlights the close relationship between psychological factors and CVD (7). Psycho-cardiology is a discipline grounded in the biopsychosocial model of medicine, which aims to prevent and treat both cardiac and psychological conditions through a holistic approach that integrates physical, psychological, and social dimensions. The historical roots of the concept can be traced back to 1628, when William Harvey proposed the circulatory system as it is understood today, and further emphasized the connection between the mind and the heart. In 1772, William Heberden provided the first detailed description of angina pectoris, noting that it could be exacerbated by mood disorders. However, for nearly two centuries, little attention was given to the potential link between psychology and heart health until a pivotal study by Frasure Smith et al. demonstrated that patients with depression during acute myocardial infarction (MI) had significantly higher mortality rates compared to those without depression (8). Since then, extensive research has been conducted on the relationship between CVD and depression, revealing that depression is highly prevalent among individuals with CVD, affecting approximately 20%–40%, and not only contributes to the onset of CVD but also worsens outcomes in patients with pre-existing heart disease (9). Today, CVD and mood disorders such as anxiety and depression are recognized as leading causes of reduced life expectancy and quality of life worldwide (10). Psychosomatic disorders like depression, have gained increasing attention within the biopsychosocial model of medicine. A current research indicates that the mechanisms underlying the coexistence of CVD and depression include autonomic dysfunction, neuroendocrine imbalance, inflammation, insulin resistance, platelet activation, and lifestyle factors (11). To further elucidate the mechanisms comprehensively, extensive research has focused on identifying potential risk factors through multi-omics data analysis (12).

The gut microbiota plays a crucial role in various aspects of human digestion, nutrient absorption, biological barrier function, immune regulation, and metabolism (13). As the largest and most complex microecological system in the human body, it comprises over 1,000 different species of bacteria, predominantly from the *Bacteroidetes*, *Firmicutes*, and *Proteobacteria* (14). Maintaining the structural and proportional balance of the community is essential for preserving gut microecological homeostasis. Advances in microbiome research have revealed that disruptions in the gut microbiota can lead to systemic diseases (15), particularly CVD and psychosomatic disorders. The stability of the gut microbiota is crucial for maintaining the physiological integrity of both the circulatory and nervous systems. On the one hand, beneficial gut probiotics produce metabolites, such as short-chain fatty acids (SCFAs), that help prevent CVD and psychosomatic disorders; on the other hand, harmful metabolites produced by certain gut bacteria can be absorbed into the circulatory system, triggering inflammatory responses and contributing to the development of systemic diseases (16).

### Gut microbiota and metabolites in CVD

Earlier sequencing studies identified bacterial DNA in human atherosclerotic plaques, but whether the DNA originated from viable bacteria within the artery wall remains inconclusive. The majority of studies on trimethylamine N-oxide (TMAO) have been pioneering in revealing the potential causal association of gut microbiome and its metabolomics with CVD. TMAO is a bioactive gut microbial metabolite produced from protein-derived foods, such as carnitine and choline found in meat and seafood, which are metabolized by gut microorganisms and subsequently oxidized in the liver. ApoE knockout mice exhibited increased aortic damage following dietary TMAO supplementation (17). *In vitro* studies have demonstrated that TMAO can stimulate platelet aggregation by enhancing calcium release from the platelet endoplasmic reticulum, thereby exerting a prothrombotic effect (18). Clinical trials have also indicated that choline supplementation can elevate fasting TMAO levels and augment platelet aggregation (19). Additionally, TMAO has been shown to activate signaling pathways in vascular smooth muscle cells and endothelial cells, leading to upregulation of inflammatory genes, increased leukocyte adhesion to endothelial cells, and elevated expression of scavenger receptors CD36 and SR-A1, thereby enhance the uptake of modified low-density lipoproteins by macrophages, subsequently promoting foam cell formation (20). Notably, Wang et al. revealed a dose-dependent association between TMAO and cardiovascular disease (CVD) (20). Furthermore, studies have demonstrated significant differences in serum TMAO concentrations among patients with varying severities of coronary atherosclerosis, and plasma TMAO may promote the progression of coronary atherosclerosis to ACS, suggesting its potential role in determining CVD severity and prognosis (21). Another study similarly demonstrated a positive correlation between TMAO levels and atherosclerotic plaque burden (22); however, the precise mechanisms underlying the association remain to be fully elucidated. With the continuous advancement of research, numerous gut microbial metabolites, such as lipopolysaccharide (LPS), SCFAs, and bile acid (BA), have been demonstrated to significantly impact the occurrence and progression of CVD by modulating inflammation, immune response, lipid metabolism, oxidative stress, and insulin resistance (23).

The relationship between the host and gut microbiota is not unidirectional but rather mutually dependent and synergistic (24). Studies have shown that patients with decompensated heart failure (HF) have higher LPS concentrations compared to those with stable HF, and the progression of HF is associated with an increase in inflammatory markers such as soluble (s) CD14, tumor necrosis factor (TNF)*α*, and interleukin 6 (IL-6) (25, 26). Multiple studies have demonstrated that HF patients experience alterations in gut barrier integrity, with elevated blood levels of pro-inflammatory cytokines correlating with symptom severity and poorer prognosis (27). A recent animal study confirmed that gut barrier function in mice was impaired following MI, leading to bacterial translocation and LPS entry into the bloodstream, which triggers inflammation and adversely affects prognosis (28). The study observed that serum LPS levels in STEMI patients peaked on the second day after symptom onset. Intriguingly, polymyxin B treatment in myocardial infarction (MI) models reduced infarct size compared to controls, implicating gut microbiota translocation and elevated LPS in post-MI inflammation and adverse cardiovascular events (28). Emerging evidence indicates that lipopolysaccharide (LPS) accelerates atherosclerotic progression through TLR4/NF-*κ*B axis-mediated upregulation of adipose differentiation-related protein (ADRP) in adventitial fibroblasts. This molecular cascade drives pathological lipid deposition and subsequent foam cell formation, a hallmark of atherogenesis (29). It is reported that SCFAs are closely linked to cardiovascular health. Animal studies indicate that SCFAs modulate blood pressure via receptors GPR41 and Olfr78. GPR41-knockout mice exhibited higher systolic blood pressure than wild-type counterparts (30). Additional research highlights SCFAs’ critical role in maintaining intestinal barrier integrity and preventing heart failure progression (31).

Disruptions in gut microbial metabolites are associated with gut dysbiosis. A meta-analysis revealed significant differences in the composition of gut microbiota between patients with coronary artery disease (CAD) and those without CAD (32). Notable distinctions included decreased levels of the genera *Bacteroides* and *Lachnospira*, alongside increased levels of the genera *Enterobacteriaceae*, *Actinobacteria*, and *Verrucomicrobia* in CAD patients. These bacterial genera are linked to altered concentrations of metabolites such as LPS, TMAO, SCFAs, and BA (32). Modulating gut microbiota dysbiosis may help reverse metabolic disorders and improve patient outcomes. In a randomized controlled trial (RCT), participants taking *Lactobacillus rhamnosus* for 12 weeks exhibited significantly reduced levels of interleukin-1 beta (IL-1β) and LPS compared to those receiving a placebo (33). Another RCT demonstrated that *Bifidobacterium lactis* supplementation for 6 months in CAD patients increased *Bacteroides* abundance, decreased *Enterobacteriaceae* levels, and reduced TMAO levels compared to placebo. Furthermore, the symptoms of angina pectoris were also significantly improved in the probiotic group (34).

The translocation of gut microbial metabolites into the circulatory system has been associated with elevated inflammatory markers and increased ischemia-reperfusion injury severity in patients with MI (35). Probiotic supplementation has demonstrated the potential to lower transforming growth factor-beta (TGF-β) and TMAO levels, suggesting therapeutic benefits for myocardial remodeling (35). Animal studies have indicated that modulating gut microbiota can restore immune cell proportions in bone marrow, reducing early inflammatory responses following MI and improving cardiac outcomes (36). In a rat model of MI, supplementation with *Lactobacillus rhamnosus* GR-1 significantly improved left ventricular ejection fraction, reduced left ventricular mass, and decreased brain natriuretic peptide levels (37). Conversely, cardiomyocyte necrosis, apoptosis, and macrophage infiltration post-MI were significantly increased in mice fed TMAO-supplemented diets (38). Additionally, chronic-phase plasma TMAO levels were associated with coronary plaque progression and increased cardiovascular event rates in patients following ST-segment elevation MI (39). Furthermore, supplementation with urolithin A, a gut microbiota-derived metabolite, was found to inhibit myocardial fibroblast transformation and improve myocardial remodeling post-MI (40). These findings underscore the potential of gut microbiota and its metabolites to enhance post-MI cardiac remodeling, providing compelling evidence for the development of gut-based therapies for CVD. In addition to CAD and MI, the composition of intestinal flora in patients with other cardiovascular diseases also changes, including atrial fibrillation, HF, and Hypertension et al. As summarized in Table 1, we have delineated the changes in gut microbiota composition associated with cardiovascular diseases.

**Table 1:** Changes of Gut Microbiota composition in different cardiovascular diseases.

Cardiovascular disease (CVD)	Increased Taxa	Decreased Taxa	References
Acute Coronary Syndrome (ACS)	Proteobacteria (phylum), Streptococcus (genus), Enterobacteriaceae (family); et al.	Faecalibacterium (genus), Bacteroides (genus); et al.	(7, 41–91)
Atherosclerosis (AS)	Clostridiaceae (family), Lactobacillus (genus); et al.	Bacteroidetes (phylum), Roseburia (genus), Faecalibacterium (genus); et al.	(7, 41–91)
Atrial Fibrillation (AF)	Megamonas (genus), Proteobacteria (phylum); et al.	Bacteroidetes (phylum), Roseburia (genus), Blautia (genus); et al.	(7, 41–91)
Coronary Artery Disease (CAD)	Proteobacteria (phylum), Lactobacillus (genus), Enterobacteriaceae (family); et al.	Lachnospira (genus), Faecalibacterium (genus), Bacteroides (genus); et al.	(7, 41–91)
Heart Failure (HF)	Proteobacteria (phylum), Streptococcus (genus), Alistipes (genus); et al.	Prevotella (genus), Roseburia (genus); et al.	(7, 41–91)
Hypertension (HTN)	Streptococcus (genus), Enterococcus (genus); et al.	Faecalibacterium (genus), Bacteroides (genus), Butyricicoccus (genus); et al.	(7, 41–91)
Stroke	Enterobacteriaceae (family), Lactobacillaceae (family), Proteobacteria (phylum); et al.	Faecalibacterium (genus), Roseburia (genus), Bacteroidetes (phylum); et al.	(7, 41–91)

In conclusion, disturbances in gut microbiota and its metabolites are associated with increased susceptibility to CVD and adversely affect prognosis. Modulation of metabolite imbalances through gut microbiota interventions holds potential for improving cardiovascular pathology. These findings provide robust evidence supporting the use of gut microbiota interventions in CVD patients; however, large-scale, long-term clinical trials are necessary to further validate their efficacy.

### Gut microbiota and metabolites in psychiatric disorders

By 1980, studies had identified polypeptide hormones produced by specific secretory cells in the gastrointestinal tract which were also present in nerve and brain cells, leading to the development of the “gut-brain axis” concept. The concept has since been evolved into the “microbiome-gut-brain axis,” highlighting the interconnections of the nervous, endocrine, immune, and inflammatory systems, along with the gut microbiota and its metabolites (92). In depressed mice, significant reduction of SCFAs in fecal samples, as well as decreases in hypothalamic 5-hydroxytryptamine and neurotransmitter levels, were closely linked to alterations in gut microbial composition (93). Another study demonstrated that gut microbes from depressed mice could induce depressive symptoms and alter gut microbiota composition in other mice (94). After ingesting the microbes, the recipient mice developed depressive symptoms and exhibited elevated levels of inflammatory markers. However, the symptoms and cytokine levels were mitigated when subdiaphragmatic vagotomy was performed, providing strong evidence for the bidirectional communication within the microbiome-gut-brain axis. A recent large-scale clinical study found that patients with depression had significantly reduced levels of *Faecalibacterium* and *Coprococcus* bacteria characterized by butyrate production, which are associated with a better quality of life (95). Butyrate, an SCFAs, helps improve gut barrier function, reduces inflammation, and promotes neurogenesis (96). A meta-analysis revealed that levels of *Faecalibacterium* and *Coprococcus* were decreased in patients with major depressive disorder, bipolar disorder, psychosis, schizophrenia, and anxiety, suggesting these diseases are characterized by a reduction in anti-inflammatory, butyrate-producing bacteria and an increase in pro-inflammatory genera (97). The finding supports the potential for gut microbiota interventions in treating psychological disorders. In a recent 6-month clinical trial, patients who received *Bifidobacterium lactis* supplementation showed significant improvements in depression and anxiety levels compared to the placebo group (34). Another systematic review highlighted that interventions with polyunsaturated fatty acids may prevent acute mood changes, inhibit inflammation, and alleviate stress-related psychological disorders such as depression and post-traumatic stress disorder (98). The underlying mechanism behind the effect may be that gut microbiota influences brain function by regulating brain areas, neurotransmitters and neuropeptides involved in mood and appetite, and probably also inpacts mood and behavior (98). While the use of probiotics and/or prebiotics for treating depression and anxiety is supported by existing evidence, there is currently a lack of corresponding clinical research. Moreover, the current understanding of the bidirectional effects of the “microbiome-gut-brain axis” is predominantly based on animal studies, with a shortage of large-scale clinical trials to confirm therapeutic efficacy.

### Gut microbiota and metabolites in psycho-cardiological disease

**Figure 1:**
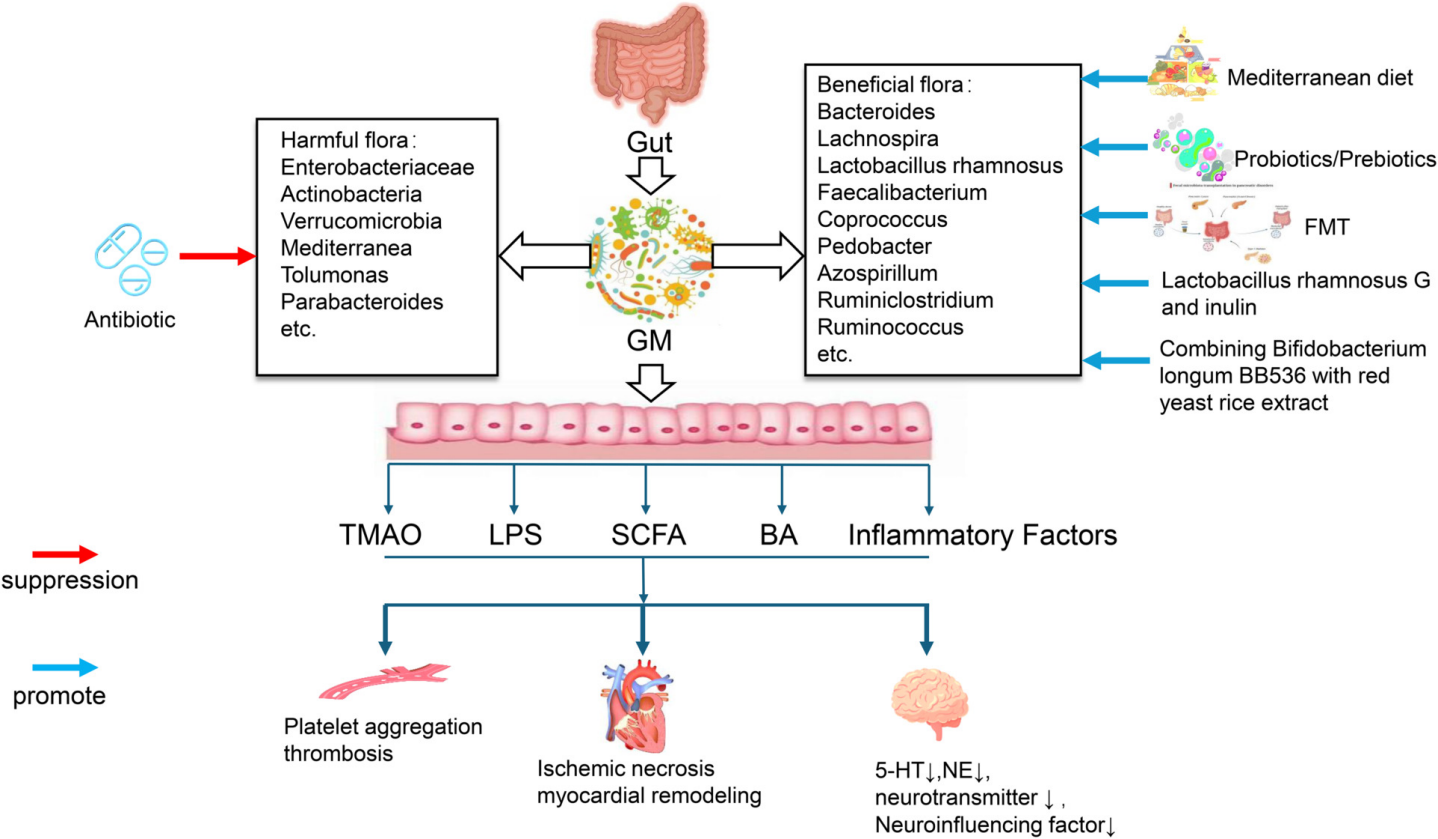
Effects of metabolites from the gut microbiota in Psycho-cardiological disease.

Given the substantial evidence linking alterations in gut microbiota and its metabolites to the pathogenesis of CVD and psychological disorders, recent basic and clinical research has increasingly focused on the role of gut microbiota in the development and prognosis of psycho-cardiological diseases. Zhang et al. conducted a comparative analysis of the gut microbiota in patients with comorbid depression and CAD, those with depression alone, and healthy individuals (99). The study found significantly elevated levels of *Enterobacterium* and *Enterococcus* in patients with both depression and CAD, while *Bifidobacterium*, *Lactobacillus*, and the *Bifidobacterium*-to-*Enterobacteriaceae* ratio (B/E ratio) were markedly lower compared to the other two groups. Moreover, logistic regression analysis identified the B/E ratio as an independent predictor of prognosis in patients with depression and CAD. In a recent cross-sectional study, fecal microbiota metagenomic and untargeted metabolomic analyses were performed on patients with heart failure (HF) and depression, patients with HF alone, and healthy individuals (100). The study revealed significant increases in the gut bacteria *Mediterranea*, *Tolumonas*, and *Parabacteroides* in patients with both HF and depression, alongside notable decreases in *Pedobacter*, *Azospirillum*, and *Ruminiclostridium*. Additionally, reduced levels of anti-inflammatory mediators (abietic acid, quinic acid, and linoleic acid) and neurotransmitters (serotonin, tryptamine, and phenylethylamine) were observed in these patients. Enrichment analysis demonstrated a strong correlation between gut microbiota and the functional pathways of metabolites, particularly those related to amino acid metabolism, fatty acid metabolism, and cAMP signaling pathways, suggesting the pathways may play a crucial role in the comorbidity of depression and HF (100). The pathophysiology of hypertension is also closely linked to psychological disorders. A novel subtype of hypertension, termed “depressive-epidemic hypertension” (DEP-HTN), was proposed in a Florida hypertension study, where individuals with DEP-HTN exhibited a distinct gut microbiota composition compared to those with either hypertension or depression alone (101). The DEP-HTN model integrates microbial taxa and functional genomics to explore the complex interactions between gastrointestinal pathophysiology and the central regulation of blood pressure and mood. Furthermore, modulating gut microbiota has shown promise in alleviating psychological symptoms in CVD patients. In an RCT, patients with CAD who received an 8-week co-supplementation of probiotics and prebiotics (*Lactobacillus rhamnosus* G and inulin) experienced significant improvements in anxiety, depression, serum high-sensitivity C-reactive protein (hs-CRP), LPS, and tumor necrosis factor-alpha levels compared to those who received a placebo (102). Notably, the combination of inulin with probiotic supplementation was more effective in improving psychological symptoms and inflammatory biomarkers than either supplement alone. Figure 1 summarized the role of the above gut microbiota metabolites in Psycho-cardiological disease. In summary, the disruption of the gut microecosystem is closely associated with the co-occurrence of CVD and psychological disorders, offering new insights into the “microbiota-gut-heart/brain axis” and presenting novel opportunities for gut microbiota-based interventions in psycho-cardiological diseases.

### Interventions targeting gut microbiota

The gut, functioning as a bridge between the body's internal and external environments, is profoundly influenced by diet. Diet can modulate the growth and activity of specific microorganisms within the gut microbiota by providing essential nutrients, thereby potentially impacting human health (103). The Mediterranean diet (MedDiet) is a predominantly plant-based dietary pattern characterized by: (a) high levels of unsaturated fatty acids, fiber, vitamins, and minerals derived from fruits, vegetables, nuts, seeds, olive oil, and whole grains; and (b) low consumption of saturated fats, meat, and dairy products (104). Numerous meta-analyses and prospective clinical trials have consistently demonstrated the cardiovascular benefits of the MedDiet (105, 106). Multi-omics studies have shown that the MedDiet is associated with the abundance of gut microbiota, such as *Faecalibacterium*, *Ruminococcus*, and *Bacteroides*, and is positively correlated with higher concentrations of SCFAs in feces (107). Additionally, several clinical trials have demonstrated that adherence to the MedDiet can effectively alleviate depressive symptoms and enhance the quality of life in patients (108, 109). Given the distinct effects of various components of the MedDiet on gut microbiota, further multidisciplinary and multi-omics research is warranted to elucidate the specific impacts of individual dietary components on gut microbial composition and function.

Compared to the complexities of dietary regulation, modulating gut microbiota through probiotics and/or prebiotics offers a more practical approach. Prebiotics are substances that stimulate microbial growth and can be fermented by gut bacteria to produce SCFAs (110), which enhance gut barrier function, and regulate metabolism, immunity, and inflammation. Additionally, prebiotics promote the growth of beneficial bacteria while inhibiting the proliferation of pathogenic bacteria (111). A clinical trial demonstrated that combining *Bifidobacterium longum* BB536 with red yeast rice extract significantly improved the atherogenic lipid profile in patients with low cardiovascular risk (112). Furthermore, after 3 months of probiotic supplementation in patients with MI, significant reductions in TGF-β, TMAO, and hs-CRP levels were observed compared to the placebo group (113). These findings suggest that probiotics may positively impact cardiac remodeling in MI patients. Another study found that patients with CAD, taking *Bifidobacterium lactis,* experienced significant improvements in angina, depression, and anxiety compared to those who received a placebo (34).

Fecal microbiota transplantation (FMT) is primarily used as a therapeutic approach for recurrent refractory *Clostridium difficile* infections (114). Recently, FMT has also been applied in the management of conditions such as ulcerative colitis and irritable bowel syndrome (115, 116). As of May 23, 2025, only four clinical trials have been registered on http://www.clinicaltrials.gov using “FMT” and “CAD” as keywords. Additionally, only twelve trials have been registered using the terms “FMT” and “Depression.” Current research on the application of FMT in psycho-cardiological disease is largely limited to animal studies. For instance, significant differences in gut microbial composition have been observed between NLRP3 knockout (KO) mice and wild-type mice, particularly in the relative abundance of *Firmicutes*, *Proteobacteria*, and *Bacteroidetes* (117). Furthermore, transplantation of gut microbiota from NLRP3 KO mice into recipient mice significantly improved depressive behaviors induced by chronic unpredictable stress (CUMS). Conversely, when gut microbiota from CUMS model mice were transplanted into normal recipient mice, higher levels of anxiety, depressive behaviors, and inflammatory factors were observed in the recipients (117).

The use of antibiotics in treating psycho-cardiological diseases has a long-established history. As early as the 1970s, tetracycline was found to readily bind to ischemic myocardial cells, and radiolabeled tetracycline was widely employed as a diagnostic tool for identifying MI areas. Minocycline, a member of the tetracycline family, has garnered significant attention as a potential therapeutic agent for CVD due to its anti-inflammatory, anti-apoptotic, and antioxidant properties (118). These beneficial effects have been demonstrated in both preclinical animal models of cardiac disease (118) and clinical trials in neurological conditions (119, 120). Additionally, research has shown that minocycline can prevent depression- and anxiety-like behaviors in animals following a stroke (119). Furthermore, hippocampal neurodegeneration was notably reduced in stroke animals treated with minocycline, suggesting that minocycline may mitigate post-stroke depression and anxiety through its neuroprotective effects following cerebral ischemia (119). However, some clinical studies have reported that minocycline does not significantly improve symptoms in patients with depression or bipolar disorder (121). Thus, further clinical research is necessary to establish the efficacy of antibiotics in treating psycho-cardiological diseases. Table 2 showed some of the above clinical trials results of interventions targeting the gut microbiota.

**Table 2:** Clinical trial results of intervention targeting Gut Microbiota for psycho-cardiological disease.

Type of trial	Intervention	Major findings	References
Randomized, crossover Study	Vegetarian diet	Of 150 eligible patients, 31 (21%) agreed to participate, and 27 (87%) participants completed the study. Mean oxidized LDL-C (−2.73 U/L), total cholesterol (−5.03 mg/dl), LDL-C (−3.87 mg/dl), and body weight (−0.67 kg) were significantly lower with the VD than with the MD. Differences between VD and MD were observed in the relative abundance of several microbe genera within the families Ruminococcaceae,achnospiraceae,and Akkermansiaceae. Plasma metabolites, including l-carnitine, acylcarnitine metabolites, and phospholipids, differed in subjects consuming VD and MD. The effect on oxidized LDL-C in response to the VD was associated with a baseline gut microbiota composition dominated by several genera of Ruminococcaceae.	(122)
Randomized controlled trial	MED,Green-MED	Both MED diets and Green-MED induced substantial changes in the community structure of the gut microbiome, with the Green-MED diet leading to more prominent compositional changes, largely driven by the low abundant,"non-core,” microorganisms. The Green-MED diet was associated with specific microbial changes, including enrichments in the genus Prevotella and enzymatic functions involved in branched-chain amino acid degradation, and reductions in the genus Bifidobacterium and enzymatic functions responsible for branched-chain amino acid biosynthesis.	(123)
Randomized, double-blind clinical trial	Co-supplementation of probiotics and inulin	Probiotic-Inulin,Co-supplementation significantly decreased BDI (−11.52 +/- 0 + 3.20 vs. + 2.97 +/- 0.39, *P* = 0.001), STAI-state (−17.63 +/- 3.22 vs. −0.60 +/- 0.33, *P* = 0.021), and STAI-trait (−24.31 +/- 7.41 vs. −1.45 +/- 0.66, *P* = 0.020) scores, hs-CRP (−1.69 +/- 0 + 66 vs. + 0.82 +/- 0.39 mg/dl, *P* = 0.020), LPS [−22.02 +/- 5.40 vs. + 0.31 +/- 0.18 (EU/L), *P* = 0.047], and TNF-alpha [−25.05 +/- 7.41 vs. + 0.79 +/- 0.71 (ng/L), *P* = 0.032] in comparison to placebo.	(124)
Observational trial	FMT,probiotic supplementation	Gut microbiota of patients with inflammatory depression exhibits higher Bacteroides and lower Clostridium, with an increase in SCFA-producing species with abnormal butanoate metabolism. After FMT, the gut microbiota of the inflammatory depression group shows increased peripheral and central inflammatory factors and intestinal mucosal permeability in recipient mice with depressive and anxiety-like behaviors. Clostridium butyricum administration normalizes the gut microbiota, decreases inflammatory factors, and displays antidepressant-like effects in a mouse model of inflammatory depression.	(125)
Double-blind,12-week, randomised, placebo-controlled trial	Minocycline,celecoxib	From baseline to week 12, depressive symptoms as per HAMD-17 reduced in all four groups (from 24.5–25.2 to 11.3–12.8), but these reductions did not differ significantly between the groups. In terms of main effects, reductions in HAMD-17 did not differ for patients treated with minocycline [mean adjusted difference vs non-minocycline 1.48 ( 95% CI −0.41 to 3.36); *p* = 0.123] or for celecoxib [mean adjusted difference vs non-celecoxib −0. 74 (−2.61 to 1.14); *p* = 0.443]. Rates of serious adverse effects did not differ between groups (31 participants had a manic switch, two self-harmed, and one died in a motor vehicle accident).	(121)

LDL-C, low-density lipoprotein cholesterol; VD, vegetarian diet; MD, meat diet; MED, Mediterranean diet; Green-MED, Green-Mediterranean diet; SCFA, short chain fatty acid; FMT, fecal microbiota transplantation; HAMD-17, hamilton depression scale.

Accumulating evidence indicates that Sodium glucose cotransporter protein inhibitor (SGLTi) plays an extremely important role in the treatment of IHD (Ischemic heart disease), and its protective effects mainly involve modulating energy metabolism, anti-inflammation, anti-fibrosis and improving the expression and function of ion channels (126). Recently, our animal study showed that sotagliflozin (SOTA), an approved sodium glucose cotransporter 1 and 2 (SGLT1/2) inhibitor for diabetes, not only improves the cardiac function of mice with MI, but also ameliorates the depression-like behaviors in the mice (127). The study also sugggestted that SOTA protected the heart mainly through regulating the composition of the gut microbiota, and found that Alloprevotella, Prevotellaceae UCG-001,and Prevotellaceae NK3B31 group may be important contributors to the SOTA treatment effects in MI mice. Meanwhile, the study further confirmed the beneficial effects of SOTA on cardiac function and depression-like behaviors in MI mice through FMT. These findings indicate that both SGLTi and FMT can ameliorate cardiac function and depression-like behaviors by modulating the gut microbiota, which provides a new direction for treating depression-like behaviors in patients with MI and promoting the recovery of cardiac function after MI. At present, however, no study has found the specific mechanism of SGLT modulating or altering the gut microbial communities of MI mice, as well as the current study is still in the animal research stage, and relevant clinical studies are also needed to evaluate the specific efficacy of SGLTi and FMT intervention on psycho-cardiologic diseases.

During the development of psycho-cardiological diseases, the reduction of some gut microbiota metabolites is closely related to the progress of the disease. Supplementing relevant gut microbiota metabolites is also a potential therapeutic strategy. Recent studies have found that supplements of native metabolites of intestinal flora is also beneficial to the treatment of double heart disease. A study found that DCA (a secondary bile acid metabolized by intestinal flora) was significantly reduced in patients with AMI by analyzing the bile acid metabolism in the serum of patients with AMI and the control group (128). The study also found that compared with the control group, the ischemic damage to cardiac function of MI mice treated with DCA was reduced, and the main mechanism was to reduce inflammation through dca-tgr5 signaling pathway to improve cardiac function after myocardial infarction. Another animal study found that TUDCA, (a bile acid, which has attracted much attention because of its protective effect on Alzheimer's disease and other brain disorders (129) reduced the increase of inflammasome and microglial activation markers in CUS mice, including interleukin- β, and nod like receptor protein 3 et al. (130). In addition, Relevant studies have proved that microglial dysfunction is closely related to the occurrence and development of neurodegenerative diseases and mental diseases (131). A prospective observational study found that SCFA producing bacteria and fecal SCFA levels in AIS patients were significantly reduced, suggesting that SCFA is a marker of the severity and prognosis of AIS, and may also be a potential therapeutic target (132). A study pointed out that transplantation of SCFA rich fecal microbiota or butyrate supplementation is an effective method for the treatment of ischemic stroke (133), but a large number of relevant clinical studies are still needed to clarify the effectiveness of this treatment strategy. In conclusion, intestinal flora plays an important role in psycho-cardiological diseases, and dysbiosis is inseparably connected with different level of microbiota metabolites. Therefore, in psycho-cardiological diseases, supplementing beneficial metabolites may also be an effective intervention. In the future, a large number of studies are needed to clarify the relevant mechanisms and therapeutic targets.

### Future perspectives

Previous studies have established a link between gut microbiota and psycho-cardiological diseases; however, the understanding of their interactions and the underlying molecular mechanisms remains limited. Additionally, many fundamental questions about the use of gut microbiota as a therapeutic intervention remain unresolved, which include identifying the specific microbial species involved, determining the optimal dosage and duration of interventions, standardizing treatment protocols, assessing the frequency of administration, evaluating potential side effects, addressing microbial dysbiosis, and elucidating the physiological mechanisms underpinning the “microbiota-gut-heart/brain axis.” Despite these gaps in knowledge, current researches indicate that metabolites produced by gut microbiota, such as SCFAs, BA, LPS, and TMAO, play significant roles in the pathogenesis of psycho-cardiological diseases. Future researches should prioritize elucidating the mechanisms and interrelationships of gut microbiota and its metabolites through both basic and clinical studies. Such efforts are essential for effectively realizing the concept of “intestinal therapy for heart and brain diseases.”
