# Supplementary figures and images for: Novel opportunity of treatment for psycho-cardiologic disease by gut microbiome
Source: Front Cardiovasc Med. 2025 Jul 22;12:1604962. doi: 10.3389/fcvm.2025.1604962 (PMC12321866; doi:10.3389/fcvm.2025.1604962)

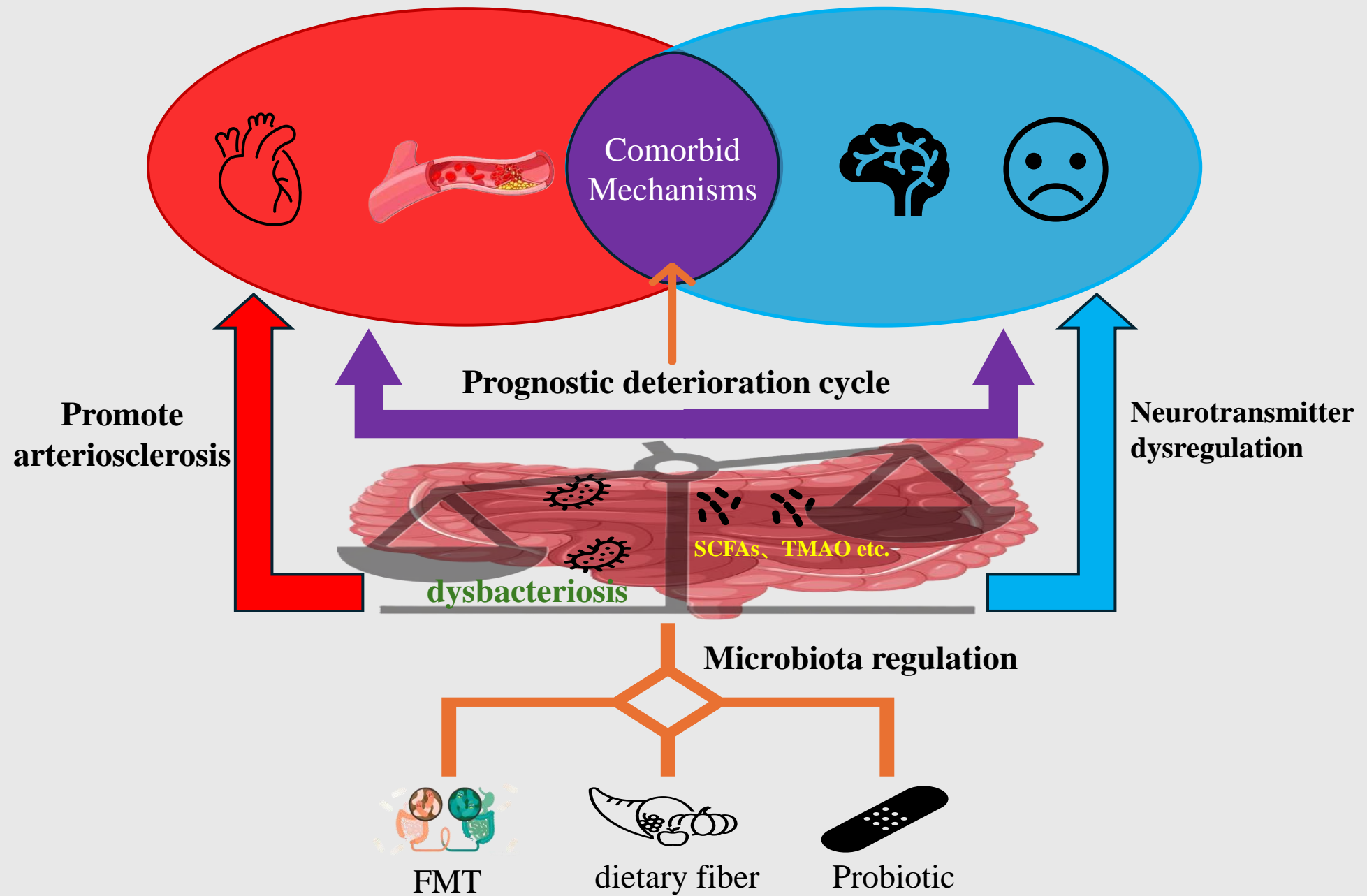

Supplement: Supplementary file 1 [file Image1.pdf]
